# Fabrication and Characterization of a Metallic–Dielectric Nanorod Array by Nanosphere Lithography for Plasmonic Sensing Application

**DOI:** 10.3390/nano9121691

**Published:** 2019-11-26

**Authors:** Yuan-Fong Chou Chau, Kuan-Hung Chen, Hai-Pang Chiang, Chee Ming Lim, Hung Ji Huang, Chih-Hsien Lai, N. T. R. N. Kumara

**Affiliations:** 1 Centre for Advanced Material and Energy Sciences, Universiti Brunei Darussalam, Tungku Link, Gadong BE1410, Negara Brunei Darussalam; chou.fong@ubd.edu.bn (Y.-F.C.C.); cheeming.lim@ubd.edu.bn (C.M.L.); roshan.kumara@ubd.edu.bn (N.T.R.N.K.); 2 Department of Optoelectronics and Materials Technology, National Taiwan Ocean University, No. 2 Pei-Ning Rd., Keelung 202, Taiwan; ethanchen74@gmail.com; 3 Institute of Physics, Academia Sinica, Taipei 115, Taiwan; 4 Taiwan Instrument Research Institute, National Applied Research Laboratories, Hsinchu 300, Taiwan; hjhuang@narlabs.org.tw; 5 Department of Electronic Engineering, National Yunlin University of Science and Technology, Yunlin 64002, Taiwan; chlai@yuntech.edu.tw

**Keywords:** periodic nanorod array, nanosphere lithography, reactive ion etching, finite element method, plasmonic sensor

## Abstract

In this paper, a periodic metallic–dielectric nanorod array which consists of Si nanorods coated with 30 nm Ag thin film set in a hexagonal configuration is fabricated and characterized. The fabrication procedure is performed by using nanosphere lithography with reactive ion etching, followed by Ag thin-film deposition. The mechanism of the surface and gap plasmon modes supported by the fabricated structure is numerically demonstrated by the three-dimensional finite element method. The measured and simulated absorptance spectra are observed to have a same trend and a qualitative fit. Our fabricated plasmonic sensor shows an average sensitivity of 340.0 nm/RIU when applied to a refractive index sensor ranging from 1.0 to 1.6. The proposed substrates provide a practical plasmonic nanorod-based sensing platform, and the fabrication methods used are technically effective and low-cost.

## 1. Introduction

Plasmonics research has shown unique absorption and enhancement of electromagnetic (EM) waves between the interface of dielectrics and metals, and more so when the metal nanostructures involved are in the subwavelength scale. Plasmon resonances occur, and these resonances are dependent on the geometrical structures of the metal nanostructures, like the localized surface plasmon resonance (SPR) and gap plasmon resonance (GPR) of metallic nanorods [1,2,3,4]. Studies have reported the potential use of these resonances, e.g., plasmonic sensors [5,6], infrared band filters [7,8], surface-enhanced Raman scattering (SERS) substrates [9,10], and heat radiation/absorption manipulation [11]. The advances in nanofabrication technologies [12,13] have a significant impact in promoting the use of plasmonic nanorod arrays as SPR sensors [14,15,16,17]. In the nanorod array configuration, these SPR sensors are highly sensitive to the changes in refractive index (RI) which influence the resonance conditions with respect to the plasmonic mode dispersion relationships [18,19,20,21,22,23,24]. In comparison to metal nanoparticle (MNP)-based configuration, the sensitivity is dependent on the near-field amplitude of the electric and magnetic fields of the localized SPRs and GPRs [25,26,27,28]. Although nanorod array-based sensing configuration possesses both SPR and GPR modes, the achieved limit of detection is still below the well-developed nanohole array surface plasmon polariton (SPP) resonance sensor [29,30,31].

A reliable fabrication technology used for the fabrication of nanorod arrays is the electron beam lithography (EBL) method. EBL is a proven technology for producing nanophotonic structures [32,33]; however, it demands highly expensive equipment and stringent fabrication conditions, consequently raising the production price. For these sensors to become commercially viable, an effective and low-priced process for fabricating metallic nanorod structures is needed. To solve this issue, various methods have been proposed, such as colloidal lithography [34], deep UV lithography [35], nanosphere lithography (NSL) [36]), reactive ion etching (RIE) [37]), chemical stamp lithography, and nanoimprinting lithography [38]. However, quality control of the process, such as the precision of size control and reproducibility must be considered. In addition, the quality control process also needs to guarantee the sensing performance of the devices. More recently, Pavlov et al. proposed a laser-printed periodically arranged nanovoid array via femtosecond (fs) laser [39], and a pattering of thin glass-supported Au films is achieved for multiple-purpose sensing platform [40,41,42]. However, an expensive femtosecond (fs) laser is required. This approach is expensive and a cheaper alternative one is required for cost-consuming. Among the recent nanofabrication techniques, the NSL and RIE methods are shown to be suitable for use with different substrates, and these technologies have a high reproducibility rate for the production of periodic array of nanorods over a large area.

In this paper, a simple and effective process to fabricate ordered metallic–dielectric Ag nanorod arrays that apply to infrared spectrum is developed and fabricated, and the feasibility study of its utilization to infrared plasmonic sensors is achieved. In order to advance the sensitivity of the nanorod array-based sensors, the coupling between the localized SPR and GPR modes were investigated. The fabrication process uses thermal evaporation in combination with NSL and RIE, to form the Si nanorod arrays with Ag coating. In the subsequent Section, the fabrication method and experimental results are clarified and explained. The diameter and height of the nanorod are shown to be highly periodic and tunable. Subsequently, the refractive index sensitivity of the plasmonic resonance and characteristic shift are investigated. A numerical model is proposed to elucidate the characteristic phenomena of the SPR and GPR mode effects arising from the fabricated nanorod array. Finally, the electric and magnetic field intensity distributions and the surface charge density distribution at the corresponding resonance wavelength (λ_res_) are calculated and analyzed. The results indicate that the fabricated nanorod array has absorptance peaks in the infrared range, and these absorptance peak wavelengths are dependent on the refractive index of the surrounding liquid under testing. The measured sensitivity observed was as high as 340 nm/RIU (where RIU denotes the refractive index unit), which is higher than existing reported experimental results [43,44,45]. The methods and results reported in this work can be also useful for the fabrication of plasmonic nanorod arrays of other metals (e.g., Au and Pt, etc.).

## 2. Fabrication Method

**Figure 1:**
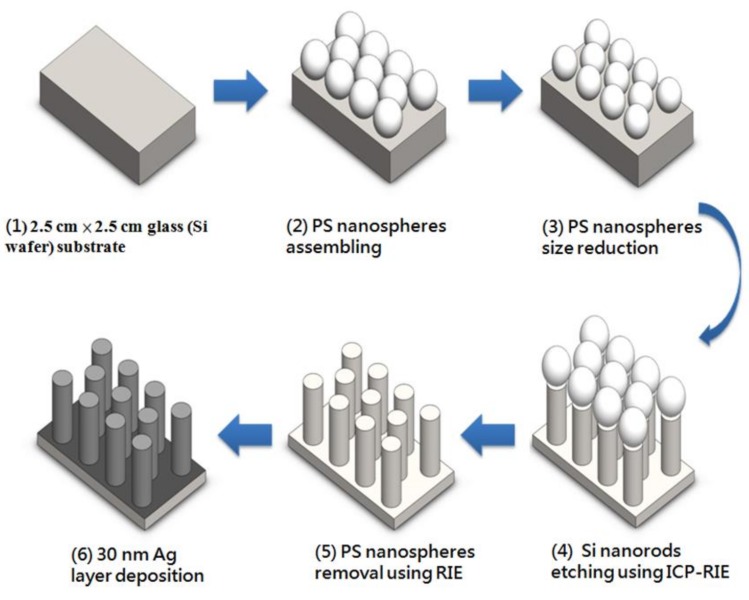
Fabrication flow of metallic–dielectric nanorod arrays on a glass (Si wafer) substrate.

The two most well-investigated plasmonic noble metals are gold (Au) and silver (Ag). In this work, Ag was chosen as a metal-deposited material, since the cost has to be taken into account. Figure 1 shows the fabrication flow of metallic–dielectric nanorod arrays on a glass (Si wafer) substrate, which includes five steps, i.e., (1) preparing 2.5 cm × 2.5 cm glass substrate, (2) polystyrene (PS) nanospheres assembling, (3) PS nanospheres size reduction, (4) Si nanorod etching, using inductively coupled plasma reactive ion etching (ICP-RIE), (5) PS nanospheres removal, using reactive ion etching (RIE), and (6) 30 nm Ag layer deposition, respectively.

**Figure 2:**
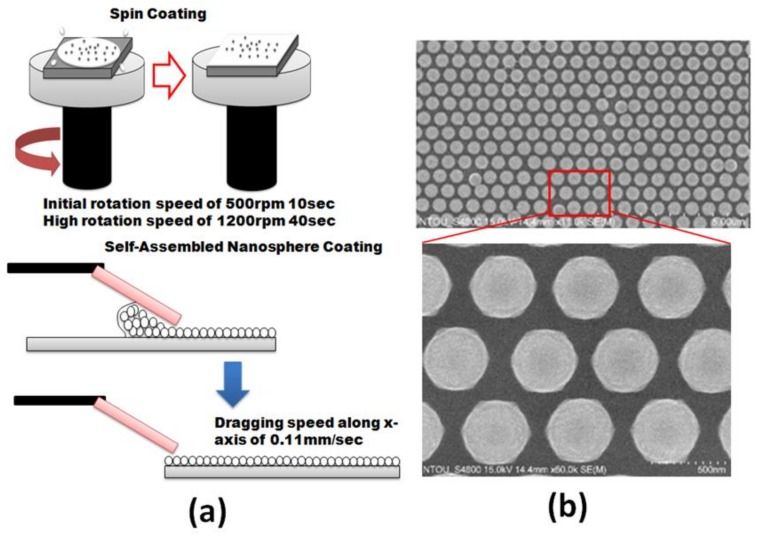
(**a**) Fabrication process of flow of self-assembled nanosphere coating and (**b**) scanning electron microscopy (SEM) images of polystyrene (PS) nanosphere assembling.

In step (1), a 2.5 cm × 2.5 cm glass (Si wafer) substrate is ultrasonically cleaned by using acetone, methanol, and distilled water in an ultrasonic bath for 20 min, respectively, and then heated on a hot plate, at 150 °C for 30 min. In step (2), a closely packed PS nanospheres monolayer is prepared by the interface method [46,47]. A layer of PS is then spin-coated onto the cleaned glass substrate (Figure 2a). The diameters of the PS nanospheres (purchased from Bangs Laboratories, Inc., PS03002, Fishers, IN, USA) are 577 nm ± 10 nm in diameter. The PS nanospheres are self-assembled by using an improved convective self-assembly method (CSA) [48]. Methanol and Triton X-100 (nonionic detergent, 400:1) are used as a surfactant. To prevent the aggregation of the PS nanospheres, the PS nanospheres are subjected to ultrasonication for 20 s. Then, the PS nanospheres are self-assembled onto the glass substrate, to form a large hexagonally ordered colloidal monolayer [49,50] (Figure 2b). In step (3), the PS nanospheres’ size reduction is performed. The dimension of the PS nanospheres is reduced by RIE technique. Oxygen plasma is used for the etching. The plasma is produced by subjecting oxygen gas to flow rate of 5 sccm, at 30 mTorr pressure, submerge in 250 W of RF power (Trion, Phantom III RIE, Tempe, AZ, USA).

**Figure 3:**
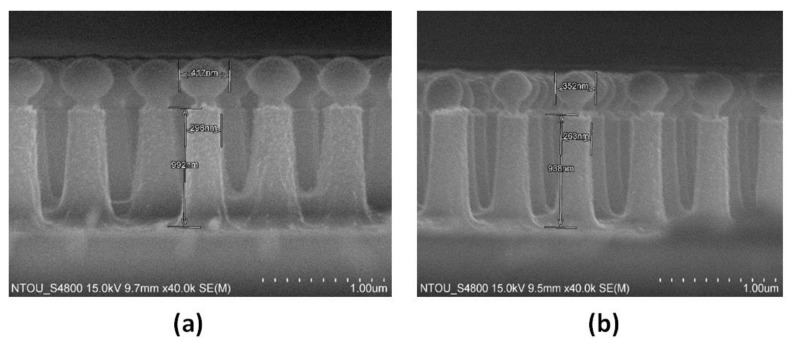
Scanning electron microscopy (SEM) images of PS nanospheres’ size reduction (**a**) after 4 min and (**b**) after 5 min, respectively.

**Figure 4:**
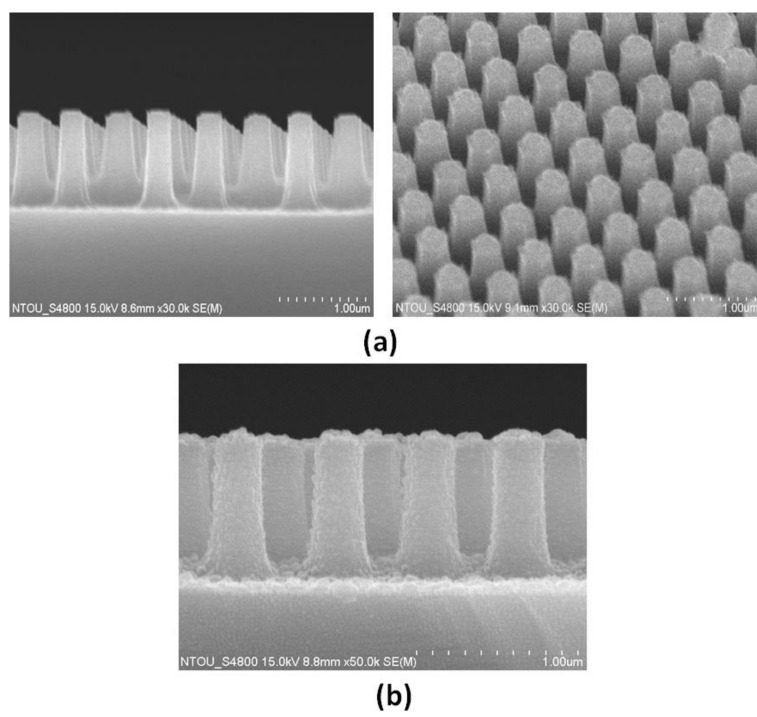
(**a**) PS nanospheres are removed by using RIE, and (**b**) a 30 nm thick Ag layer is deposited vertically onto the surface of the Si nanorods, respectively.

In step (4), we process the Si nanorod etching by using ICP-RIE. The resulting template is heated on a hot plate, at 95 °C, to allow the PS nanospheres to be embedded on the Si nanorods via capillary wetting. The height (*h*) and diameter (*d*) of the Si nanorod and the diameter (*d_1_*) of the PS nanosphere can be modified by changing the different RIE/ICP-RIE etching time. Figure 3a,b shows the scanning electron microscopy (SEM) images of PS size reduction after 4 and 5 min, respectively. The comparison of different RIE/ICP-RIE etching times of the PS nanosphere is illustrated in Table S1 (see Supplemental Material). After cooling to room temperature, wet etching with an etchant (Hydrofluoric acid) for 5 min is used to remove the PS nanospheres from the top of Si nanorods, and this leaves a metallic–dielectric nanorod array (Figure 4a). The template is then flushed several times with nitrogen gas, and it is kept in a fume hood, to allow for total evaporation of the solvent.

In step (5), PS nanospheres are removed by using RIE (see Figure 4a). In the final step, a 30 nm thick Ag layer is deposited vertically onto the surface of the Si nanorods, leading to the formation of a periodic nanorod array (Figure 4b). The merit of the Ag coating over the Si nanorods is to support a steady attachment between the Ag nanoparticles and the Si nanorods. A 30 nm thick Ag film is deposited with a deposition rate of 4 Ǻ/s by using thermal evaporation under the pressure of 5 × 10^−6^ Torr.

## 3. Measurement and Simulation Methods

In the experiment, the reflectance (R) and transmittance (T) are measured by utilizing a spectrometer (PerkinElmer Lambda-1050, Akron, OH, USA) equipped with a 160 mm integrating sphere. The measured absorptance (A) is obtained by subtracting the sum of normalized reflectance and transmittance from unity.

**Figure 5:**
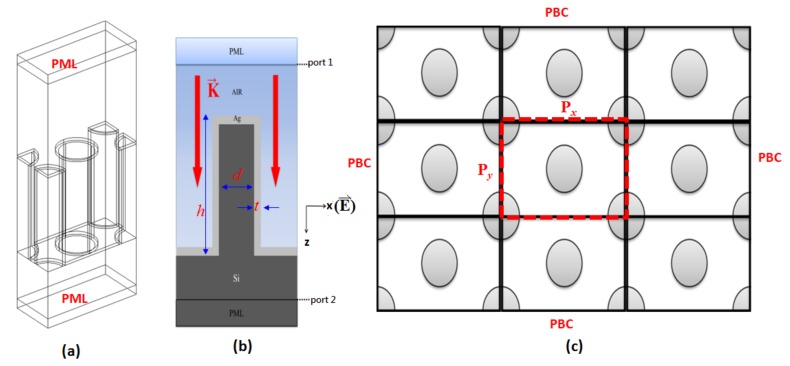
(**a**) The unit cell of simulation model, (**b**) the side view of the central nanorod in the unit cell (i.e., at the middle plane of central nanorod), and (**c**) the top view of the periodic structure, respectively. Where the enclosed red line in (**c**) indicates the region of a unit cell.

In the simulations, the unit cell of the simulation model is shown in Figure 5a, and the side view of the central nanorod in the unit cell and the top view of the periodic structure are also shown in Figure 5b,c, respectively. The enclosed red line in Figure 5c indicates the top view of a unit cell. The structural parameters are set to be *P_x_* (period along *x* axis), *P_y_* (period along *y* axis), *d* (diameter of Si nanorod), *t* (thickness of Ag layer), and *h* (height of nanorod), respectively. The absorbance spectra are calculated by using three-dimensional (3-D) finite element method (FEM) (Comsol Multiphysics [51]). The absorbance spectrum is obtained from scattering parameters (S-parameter), i.e., A (absorptance) = 1 − R(reflectance) − T(transmittance). S-parameter can be defined as S_11_ and S_21_; where S_11_ = [(power reflected from port 2)/(power incident on port 1)]^1/2^ and S_21_ = [(power reflected from port 2)/(power incident on port 1)]^1/2^, respectively. The incident light from port 1 and port 2 is a receiver plane (see the inset of Figure 5b). Reflectance and transmittance are calculated by |S_11_|^2^ and |S_21_|^2^, respectively. The Ag permittivity data is obtained from [52]. The dielectric constant of Si is obtained from [53]. A plane wave polarized in *x* axis is used as the incident light at normal incidence from the top surface (port 1). The simulation zone is divided into the tetragonal meshes, terminated by perfectly matched layer (PML) boundaries on the top and bottom sides. To mimic the periodic array of the fabricated structures, a periodic boundary condition (PBC) is set to be surrounded the side walls of the simulation region.

## 4. Results and Discussion

**Figure 6:**
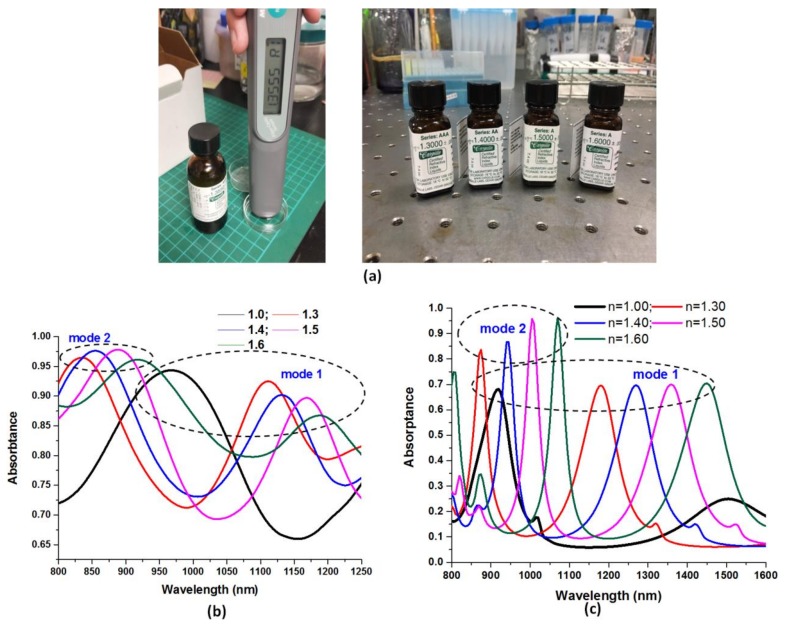
(**a**) Measured liquid solution of different refractive index (*n* = 1.3, 1.4, 1.5, and 1.6) used in the experiment. (**b**) Measured and (**c**) simulated absorptance spectra of the fabricated structures under testing at different surrounding medium (i.e., *n* = 1.0, 1.3, 1.4, 1.5, and 1.6). The structural parameters, *h*, *P_x_*, *P_y_*, *d,* and *t*, are 1000, 1030, 470, 270, and 30 nm, respectively.

The electric and magnetic field enhancements are mainly arisen from the superposition of the incident EM wave with the reflected EM wave at the boundary of the dielectric (air) and metal inter-surface and among the metallic–dielectric nanorod structures. This phenomenon is important for the design of plasmonic sensor where larger electric and magnetic field intensities are desired. Figure 6a shows the measured liquid solution of different refractive indices (*n* = 1.3, 1.4, 1.5, and 1.6) used in the experiments. The structural parameters, *h*, *P*_x_, *P*_y_, *d,* and *t*, are 1000, 1030, 470, 270, and 30 nm, respectively. A numerical prediction of the spectral response, based on FEM, is compared to the experimental observations. The illustrations in Figure 6b,c depict the absorptance spectra of the fabricated structure under testing in various surrounding medium (i.e., *n* = 1.0, 1.3, 1.4, 1.5, and 1.6), respectively. In Figure 6b,c, there are two available modes (denoted by mode 1 and mode 2) for sensing application, and the measured and simulated absorptance spectra are observed to have a same trend and a qualitative fit in the wavelength range of mode 1 and mode 2, as indicated by the insets of Figure 6b,c (enclosed by dashed black lines), respectively. From Figure 6b,c, it is evident that the absorptance peaks shifted to the longer wavelength (i.e., redshift) when the refractive index of the liquid is increased. This is considered to be owing to the coupling of the localized SPR and GPR modes with the evanescent modes. This characteristic is useful in plasmonic sensors. This technology can be also utilized for the sensing of biomolecules, such as viruses and protein.

The line shape of the absorptance spectra obtained from the simulations (Figure 6c) shows a liner relationship because the structural parameters in the simulations are the same in each unit cell. The simulation results obtained from different aspect ratios of nanorods also displayed the same trend (e.g., Figures S1 and S2 in Supplemental Material). In reality, the discrepancy between measured and simulated results is inevitable because the uniformity of the structure array could play a key role on the sensitivity performance. The nanometer scale surface roughness can significantly affect the performance of gap plasmon-based structures [54]. For example, it can be observed in Figure 4b that the roughness of Ag-deposited nanorods is revealed. In such an experimental case, localized surface plasmon can be excited at the rough surfaces [55], resulting in a difference plasmonic mode compared to the simulated one with a smooth surface. This drawback can be overcome if the GPR effect is much higher than the SPR effect in the proposed structure [56,57]. There are various factors to explain the differences, e.g., fabrication tolerances, measurement limitations, uniformity of coating Ag film, concentration of liquid solution, roughness of metal-deposited rods, Cassie–Baxter hydrophobic state of metal-deposited rods (which can be examined by contact-angle measurements) [58,59], and diverse external disturbances. In addition, the difference between the measured and simulated results can be effectively reduced by improving the uniformity and abovementioned factors in the experiments. In this case, the measured sensitivity (S), figure-of-merit (FOM) and quality (Q) factor in mode 1 of the fabricated structure can reach as high as 315.82 nm/RIU, 6.48 RIU^−1^, and 10.09, respectively. This indicates that the fabricated nanorod structure is very sensitive to the change of the surrounding medium under testing. Nanoscale surface corrugation strongly determines the plasmonic response of coated Ag-deposited nanorods with dimensions of several tens of nanometers, which verifies the potential role of nanoscale surface texturing on the plasmonic response of coated Ag-deposited nanorods. The sensitivity performance of the experiments degrades crucially depending on the nonuniformity in the fabrication process [60,61,62,63].

**Figure 7:**
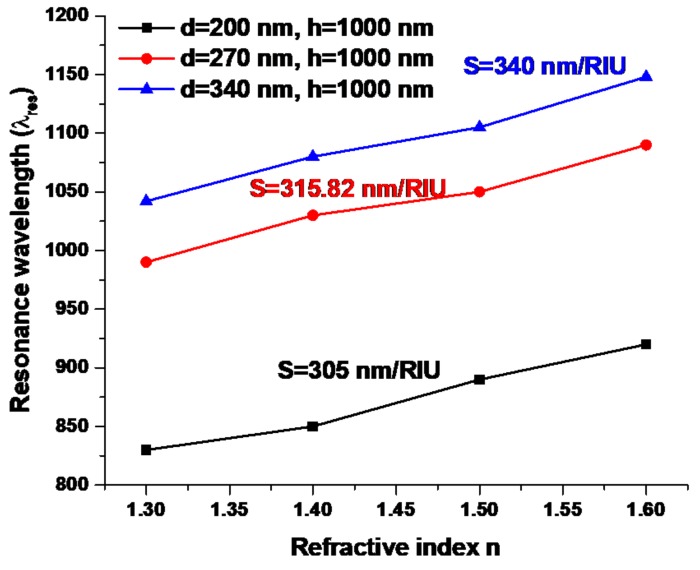
Resonance wavelength versus refractive index, where RIU represents the refractive index unit. The diameter of Si nanorod is varied from *d* = 200 to 270 to 340 nm, respectively, while the other structural parameters, *h*, *P*_x_, *P*_y_, and *t*, are kept at 1000, 1030, 470, and 30 nm, respectively.

An infrared spectrum of the nanorod array with required absorptance peak wavelength can be obtained by tuning the dimension of metallic–dielectric nanorod. By using this nanorod filter, the signal-to-noise ratio can be properly decreased and the target signal can be successfully measured. In addition, the fabricated metallic–dielectric nanorod array can be applied as an absorber of heat radiation energy [43]. Figure 7 shows the experimental results of different size of fabricated metallic–dielectric nanorod structure versus the resonance wavelength at the different refractive index of the dropped liquid under testing. The diameter of Si nanorod is varied from *d* = 200 to 270 to 340 nm, respectively, while the other structural parameters, *h*, *P*_x_, *P*_y_, and *t*, are kept at 1000, 1030, 470, and 30 nm, respectively. The measured sensitivities are also shown in the inset of the figure, and these results are remarkably higher than those of previous reported experimental works [43,44,45,64,65,66,67]. In addition, Table S2 (see Supplemental Material) also shows the comparison of sensing performance for different reported plasmonic nanosensors in the literature [68,69,70,71,72,73,74,75,76]. The sensitivity indicates the change in the peak wavelength of the absorbance spectrum versus the change in the refractive index of the dropped liquid. From Figure 7, it is found that the peak wavelength linearly increases with the dropped liquid’s refractive index. The measured sensitivities in mode 1 are 305.7, 315.8, and 340.0 nm/RIU, respectively. The gap distance will be reduced as the diameter of Si nanorod (*d*) is increased, while the other structural parameters are intact. The refractometric sensitivity is shown to be enhanced upon increasing the *d* value (i.e., decreasing the gap distance) because of an increased plasmonic effect formed in the gap region and dipolar effect occurred on the metal surface. Based upon the nanorod gap distance, this splitting of the mode would be fairly symmetrical due to dipolar interactions being dominant [77]. It can be demonstrated that the sensitivity is significantly influenced by the Si nanorod diameter, *d*, which decides the gap distance among the nanorods, and this, in turn, results in different resonant conditions of the SPR and GPR modes. In addition, the etching effect associated with different RIE/ICP-RIE etching time has a significantly impact on the influence of the structure uniformity and sensitivity performance [54,55].

**Figure 8:**
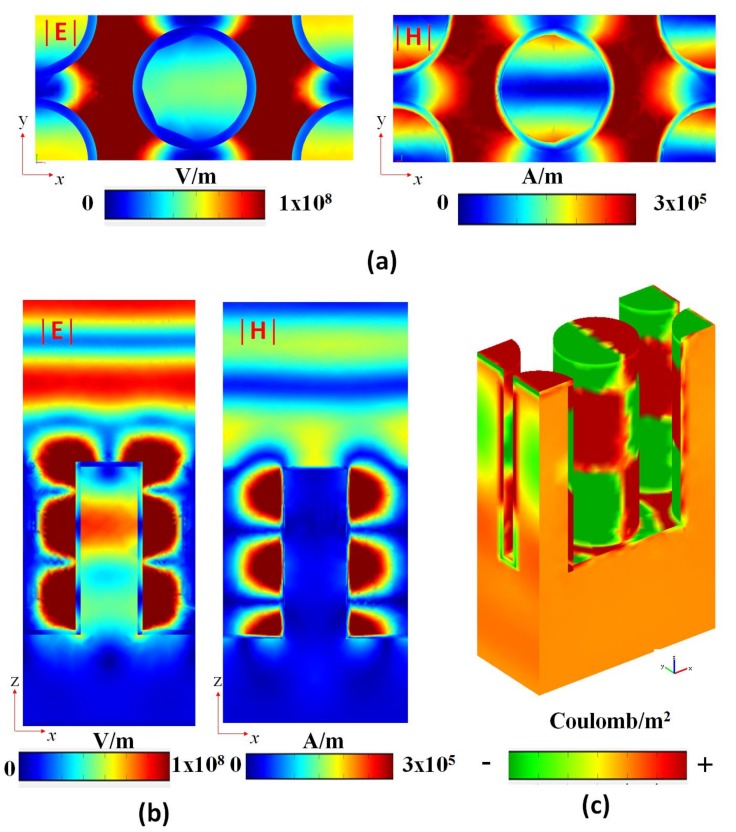
Electric field intensity at the middle plane of nanorods in x-y plane (|***E***|, left panel of (**a**) and (**b**), magnetic field intensity at the middle plane of central nanorod in x-z plane (|***H***|, right panel of (**a**) and (**b**)), and (**c**) the surface charge density distributions at the corresponding λ_res_ of 910 nm in air surrounding case (*n* = 1.0). The structural parameters, *h*, *P_x_*, *P_y_*, *d,* and *t*, are 1000, 1030, 470, 270, and 30 nm, respectively.

To further comprehend the physical mechanism, we calculate the electric field intensity (|***E***|, left panels in Figure 8a,b, magnetic field intensity (|***H***|, right panels in Figure 8a,b), and the surface charge density distribution (Coulomb/m^2^, Figure 8c) at the corresponding λ_res_ of 910 nm under air surrounding (i.e., *n* = 1.0). In Figure 8, the structural parameters, *h*, *P*_x_, *P*_y_, *d,* and *t*, are 1000, 1030, 470, 270, and 30 nm, respectively. It is obvious that the intensity distribution of |***E***| and |***H***| at resonance wavelength is highly trapped among the gap region (i.e., gap plasmon resonance [77]) and the edge surface (i.e., edge enhancement [78]), which are induced by constructive interference, and this effect could enhance the absorptance [79]. The |***E***| distribution shows an enhanced distribution profile both on the top and side surfaces, while the |***H***| distribution appears on the side surface. Note that the less |***E***| and |***H***| distributions in the core region of the nanorod is due to the skin depth of Ag thin film.

Taking into account the device’s sensing performance, it is demonstrated that the Mie-resonant structures provide limited precision in comparison to dark modes [80,81] and non-radiation charge–current configurations [82,83]. As is well-known, the electric dipoles are produced by the surface charge pairs and form the dipole moments between the gaps and metal surfaces. The origin of |***E***| and |***H***| distributions in Figure 8a,b can be interpreted by the surface-charge density distributions, as shown in Figure 8c. It is well-known that the surface-charge density (i.e., Coulomb/m^2^) can be increased by the accumulation of positive–negative charge pairs on the metal surface. These surface charges exhibit a typical dipole-like charge pattern, whose resonance is governed by the combination of SPR and GPR modes (i.e., hybrid plasmon mode [84,85,86,87,88]). This gives rise to a stronger dipolar effect and enhances the field pattern around the gap and edge regions. In Figure 8c, the surface-charge pairs from top to bottom of the surface distribute strongly and uniformly in the arrangement of (− +) (+ −) (− +) in the central part of nanorod, (+ − +) in the left side of nanorod, (− + −) in the right side of nanorod, and (+ − + − + −) on the bottom surface of Ag thin film, respectively. In these cases, the contributions of the SPR and GPR modes are remarkable contributed by the dipolar effects of the nanorod surface and gap regions. This can be verified by the distribution profiles of |***E***| and |***H***|, which show stronger field patterns at the edge surface and in the gap regions.

## 5. Conclusions

In this paper, we demonstrate experimental and numerical studies on a periodic metallic–dielectric nanorod array that can realize plasmonic sensor application in the near-infrared region. The nanorod array structure with Ag coating fabricated by a combination of nanosphere lithography with reactive ion etching (RIE), followed by metallic thin film deposition, was verified to be relatively cost-effective and highly reproducible over a large area. Furthermore, the fabrication technique is configurable to tune the performance of the plasmonic nanosensor. The measured spectra and the numerically generated spectra are shown to have a same trend and a qualitative fit in the near-infrared. The influence of the mechanisms of electric and magnetic field intensities and surface charge density on the sensing performance was also investigated, to demonstrate the capability of the proposed composite nanoresonant SPR and GPR array sensor, which showed pronounced improvement in the limit of detection compared to conventional nanorod array sensing configurations. Experimental results indicate that the fabricated metallic–dielectric nanorod array has absorptance peaks in the infrared range, and these absorptance spectra are significantly influenced by the dimension of the nanorod and the refractive index of the surrounding liquid under testing. The fabricated plasmonic nanosensor shows an average sensitivity of 340.0 nm/RIU over the refractive index sensing in the range from 1.0 to 1.6, which is remarkably higher than those of reported experimental results. The proposed substrates are potential for a variety of nanorod-array-based detection applications, from nanophotonics to biophysics.

## Supplementary Materials

The following are available online at https://www.mdpi.com/2079-4991/9/12/1691/s1. Table S1: Comparison of different RIE/ICP-RIE etching time of PS sphere. Figure S1: Simulated absorptance spectra of the fabricated structures at different surrounding medium (h = 500) and Figure S2: Simulated absorptance spectra of the fabricated structures at different surrounding medium (h = 1000). Table S2: Comparison of the current plasmonic nanosensor with the reported plasmonic sensors.
