# Supplementary material for: Fabrication and Characterization of a Metallic–Dielectric Nanorod Array by Nanosphere Lithography for Plasmonic Sensing Application
Source: Nanomaterials (Basel). 2019 Nov 26;9(12):1691. doi: 10.3390/nano9121691 (PMC6956078; doi:10.3390/nano9121691)
Supplement: Supplementary file 1 [file nanomaterials-09-01691-s001.pdf]

nanomaterials

Supplementary Information

Fabrication and Characterization of a Metallic–Dielectric Nanorod Array by Nanosphere Lithography for Plasmonic Sensing Application

Yuan-Fong Chou Chau [1], Kuan-Hung Chen [2], Hai-Pang Chiang [2,3,*], Chee Ming Lim [1], Hung Ji Huang [4], Chih-Hsien Lai [5] and N. T. R. N. Kumara [1]

[1] Centre for Advanced Material and Energy Sciences, Universiti Brunei Darussalam, Tungku Link, Gadong BE1410, Negara Brunei Darussalam; chou.fong@ubd.edu.bn(Y.-F.C.C.); cheeming.lim@ubd.edu.bn (C.M.L.); roshan.kumara@ubd.edu.bn (N.T.R.N.K.)

[2] Department of Optoelectronics and Materials Technology, National Taiwan Ocean University, No. 2 Pei-Ning Rd., 202 Keelung, Taiwan; ethanchen74@gmail.com (K.-H.C.)

[3] Institute of Physics, Academia Sinica, Taipei 115, Taiwan

[4] Taiwan Instrument Research Institute, National Applied Research Laboratories, 300, Hsinchu, Taiwan; hjhuang@narlabs.org.tw (H.J.H.)

[5] Department of Electronic Engineering, National Yunlin University of Science and Technology, Yunlin, 64002, Taiwan; chlai@yuntech.edu.tw (C.-H.L.)

* Correspondence: hpchiang@mail.ntou.edu.tw (H.-P.C.); Tel.: +886-2-24622192#6702

1. Co.mparison of different RIE/ICP-RIE etching time of PS sphere

Table S1. Comparison of different RIE/ICP-RIE etching time of PS sphere. The height (*h*) and diameter (*d*) of Si nanorod and diameter (d_1) of PS nanosphere can be modified by changing the different RIE/ICP-RIE etching time.

PS sphere RIE/ICP reaction time		RIE (3 min.)	RIE (4 min.)	RIE (5 min.)	RIE (6 min.)
ICP RIE (3 min.)	*h*	903±45.2 nm	868±50.5 nm	878±53.8 nm	912±42.5 nm
	d	350±23.6 nm	300±21.4 nm	250±20.6 nm	200±18.5 nm
	d_1	325±26.3 nm	275±27.6 nm	215±25.6 nm	149±23.0 nm
ICP RIE (3 min. 30 sec)	*h*	969±46.8nm	959±34.6 nm	923±52.1 nm	946±49.3 nm
	d	350±30.0 nm	300±28.5 nm	250±24.2 nm	200±23.7 nm
	d_1	342±23.5 nm	255±18.5 nm	260±16.3 nm	159±16.8 nm
ICP RIE (4 min.)	*h*	1200±25.5nm	1030±38.4 nm	989±49.6 nm	1100±53.5 nm
	d	350±25.3 nm	300±23.5 nm	250±19.4 nm	200±16.3 nm
	d_1	341±28.3 nm	242±25.4 nm	205±23.2 nm	189±18.6 nm

2. Si.mulated absorptance spectra of the fabricated structures at different surrounding medium

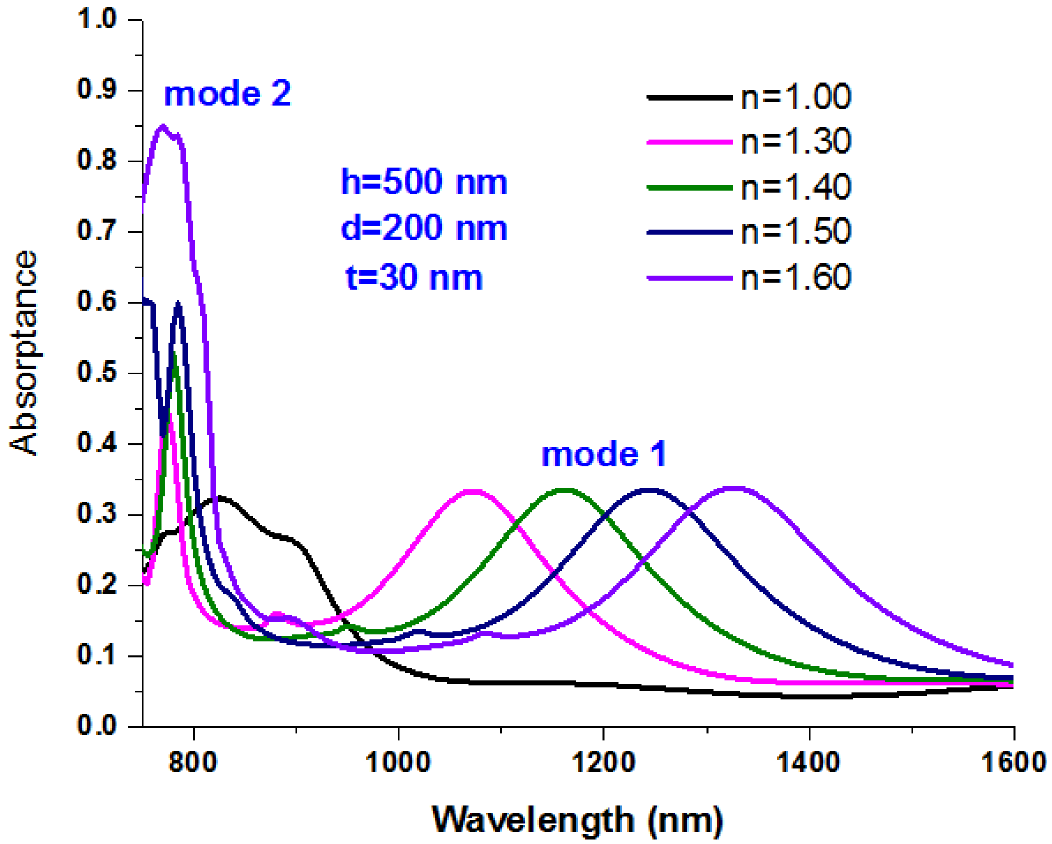

Figure S1. Simulated absorptance spectra of the fabricated structures at different surrounding medium (i.e., n=1.0, 1.3, 1.4, 1.5 and 1.6). Where h, d and t represent height and diameter of Si nanorod and thickness of Ag film, respectively.

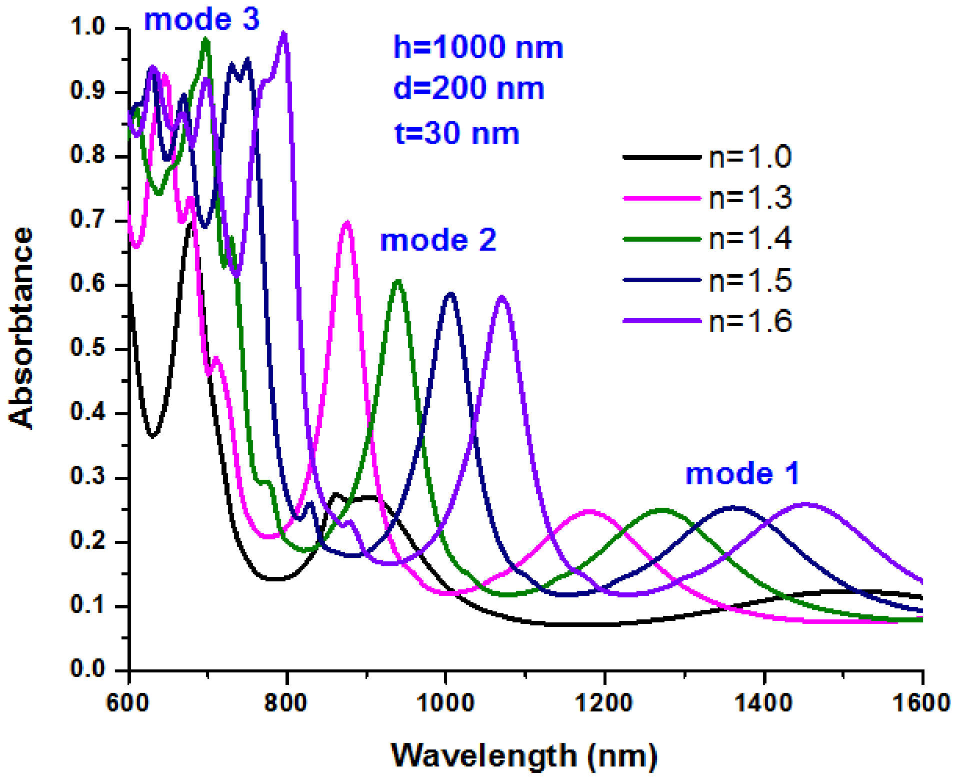

Figure S2. Simulated absorptance spectra of the fabricated structures at different surrounding medium (i.e., n=1.0, 1.3, 1.4, 1.5 and 1.6). Where h, d and t represent height and diameter of Si nanorod and thickness of Ag film, respectively.

3. Co.mparison of the current plasmonic nanosensor with the reported plasmonic sensors

Table S2 Comparison of the current plasmonic nanosensor with the reported plasmonic sensors.

Ref.	Sensing type	Targeted object	Detection limitation	Sensitivity(S)	Year
[9]	Plasmonic Nanocavities	Dispersionless material	-	~541nm/ RIU (simulation)	2008
[3]	Metallic dot	Liquid solution	-	-	2015
[5]	Plasmonic silver nanorods	Liquid solution	RI 1.3~1.4	270 nm/ RIU (experiment)	2011
[4]	Gold nanodisk	Liquid solution	RI 1.32~1.42	167-327 nm/ RIU(experiment)	2011
[2][13]	Nano hole	Gas	RI 1~1.4	600 nm/ RIU	2012
[8]	All-semiconductor plasmonic gratings	Dispersionless material	-	~900±20nm/ RIU	2016
[6]	Nanoplasmonic structure	Amide I/Monomers/Fibrils and Oligomers	-	220 nm/ RIU (experiment)	2017
[12]	Optical fiber	Aqueous solutions	RI 1.33 ~ 1.3657	700.3 nm/RIU (experiment)	2017
[7]	Gold nanoantenna array	Glucose and fructose solutions	55 mM	10 g/l (experiment)	2019
[10]	Metal Dichalcogenide	Aqueous solutions	-	198∘C/RIU (simulation)	2019
[1]	Ordered Metal Nanodot Arrays	Liquid solution	-	310 nm/ RIU (experiment)	2019
[11]	Au nanodisk array	Gas	RI 1~1.04	2262 nm/ RIU (simulation)	2019
This work	Ordered Matal nanorod array	Liquid solution	RI 1.30 to 1.60	340 nm/ RIU (experiment) 1000 nm/ RIU (simulation)	2019

References

1. Yoshino, M., Kubota, Y.; Nakagawa, Y.; Terano, M.; Efficient Fabrication Process of Ordered Metal Nanodot Arrays for Infrared Plasmonic Sensor. Micromachines **2019**, *10*, 385.
2. Im, H.; Sutherland, J.N.; Maynard, J.A.; Oh, S.H. Nanohole-based surface plasmon resonance instruments with improved spectral resolution quantify a broad range of antibody-ligand binding kinetics. Anal Chem **2012**, *84*, 1941–1947.
3. Yoshino, M.; Li, Z.; Terano, M. Theoretical and experimental study of metallic dot agglomeration induced by thermal dewetting. ASME J. Micro Nano-Manuf. **2015**, *3*, 021004.
4. Lee, S.; Lee, K.; Ahn, J.; Lee, J.; Kim, M.; Shin, Y.-B. Highly sensitive biosensing using arrays of plasmonic Au nanodisks realized by nanoimprint lithography. ACS Nano **2011**, *5*, 97–904.
5. Jakab, A.; Rosman, C.; Khalavka, Y.; Becker, J.; Tru¨gler, A.; Hohenester, U.; So¨nnichsen, C. Highly Sensitive Plasmonic Silver Nanorods. ACS Nano **2011**, *5*, 6880–6885.
6. Etezadi, D.; Warner, J.B.; Ruggeri, F.S.; Dietler, G.; Lashuel, H.A.; Altug, H. Nanoplasmonic mid-infrared biosensor for in vitro protein secondary structure detection. Light Sci. Appl. 2017, 6, e17029.

7. Kuhner, L.; Semenyshyn, R.; Hentschel, M.; Neubrech, F.; Tarin, C.; Giessen, H. Vibrational sensing using infrared nanoantennas: Toward the noninvasive quantitation of physiological levels of glucose and fructose. ACS Sens. **2019**, *4*, 1973.
8. F. B.; Barho, F. Gonzalez-Posada, M.-J. Milla-Rodrigo, M.; Bomers, L. Cerutti, and T. Taliercio, All-semiconductor plasmonic gratings for biosensing applications in the mid-infrared spectral range, Opt. Express **2016**, *24*, 16175.
9. Hao, F.; Sonnefraud, Y.; Dorpe, P.V.; Maier, S.A.; Halas, N.J.; Nordlander, P. Symmetry Breaking in Plasmonic Nanocavities: Subradiant LSPR Sensing and a Tunable Fano Resonance. Nano Lett. **2008**, *8*, 3983–3988.
10. Yi Xu, Yee Sin Ang, Lin Wu and Lay Kee Ang, High Sensitivity Surface Plasmon Resonance Sensor Based on Two-Dimensional MXene and Transition Metal Dichalcogenide: A. Theoretical Study, Nanomaterials **2019**, *9*, 165.
11. Alharbi, R.; Mustafa, Yavuz, M. Promote Localized Surface Plasmonic Sensor Performance via Spin-Coating Graphene Flakes over Au Nano-Disk Array. Photonics 2019, 6, 57.
12. Jiang, S.; Li, Z.; Zhang, C.; Gao, S.; Li, Z.; Qiu, H.; Li, C.; Yang, C.; Mei, L.; Liu, Y. A novel U-bent plastic optical fibre local surface plasmon resonance sensor based on a graphene and silver nanoparticle hybrid structure. J. Phys. D. Appl. Phys. **2017**, *50*, 165105.
13. Jianga, J.; Wanga, X.; Li, S.; Ding, F.; Li, N.; Meng, S.; Li, R.; Jia Qi, J.; Liu, Q.; Liu, G.L. Plasmonic nano-arrays for ultrasensitive bio-sensing. Nanophotonics 2018, 7, 1517–1531.
